# Supplementary material for: Reconciling Mining with the Conservation of Cave Biodiversity: A Quantitative Baseline to Help Establish Conservation Priorities
Source: PLoS One. 2016 Dec 20;11(12):e0168348. doi: 10.1371/journal.pone.0168348 (PMC5173368; doi:10.1371/journal.pone.0168348)
Supplement: S1 Dataset — (ZIP) [file pone.0168348.s002.zip › Taxa/Serra Norte/SN_2013/N4WS/Lista N4WS-77.pdf]

CAVIDADE N4WS-0077

Classe	Ordem	Fam/Outros	Gên/Outros	Espécie	S-U	U-S	Zon.
Annelida	Oligochaeta			sp.1	3		E
Arachnida	Acari	Erythraeidae		sp.1	X		E
Arachnida	Araneae	Filistatidae		sp.1	X		E
Arachnida	Araneae	Oonopidae	*Neoxyphinus*	sp.1		X	E
Arachnida	Araneae	Pholcidae		jovem		X	E
Arachnida	Araneae	Prodidomidae	*Lygromma*	sp.1	X		E
Diplopoda	Polyxenida	Hypogexenidae		sp.1	X		E
Entognatha	Collembola	Entomobryidae		sp.6		X	E
Entognatha	Diplura	Campodeidae		sp.1	X		E
Insecta	Blattodea			jovem	2		E
Insecta	Coleoptera	Tenebrionidae		jovem	X		E
Insecta	Diptera	Cecidomyiidae	Cecidomyiinae	sp.	X		E
Insecta	Diptera	Tipulidae	Tipulinae	sp.	X		E
Insecta	Homoptera	Cixiidae		jovem		X	E
Insecta	Hymenoptera	Formicidae	*Acromyrmex*	sp.1		X	E
Insecta	Hymenoptera	Formicidae	*Gnamptogenys*	*striatula*	X		E
Insecta	Lepidoptera	Noctuoidea		sp.1		8	E
Insecta	Orthoptera	Phalangopsidae	*Paraclodes*	sp.	1		E
Amphibia	Anura	Strabomantidae	*Pristimantis*	cf. *fenestratus*	1	2	E
Mammalia	Chiroptera	Emballonuridae	*Peropteryx*	sp.	2	2	E
